# Cooperation Not Competition: Bihemispheric tDCS and fMRI Show Role for Ipsilateral Hemisphere in Motor Learning

**DOI:** 10.1523/JNEUROSCI.3414-16.2017

**Published:** 2017-08-02

**Authors:** Sheena Waters, Tobias Wiestler, Jörn Diedrichsen

**Affiliations:** ^1^Institute of Cognitive Neuroscience,; ^2^Institute of Neurology, and; ^3^Sobell Department for Motor Neuroscience and Motor Disorders, University College London, London WC1 3AR, United Kingdom, and; ^4^Brain and Mind Institute, Western University, London, Ontario N6A 5B7, Canada

**Keywords:** interhemispheric communication, neurostimulation, plasticity

## Abstract

What is the role of ipsilateral motor and premotor areas in motor learning? One view is that ipsilateral activity suppresses contralateral motor cortex and, accordingly, that inhibiting ipsilateral regions can improve motor learning. Alternatively, the ipsilateral motor cortex may play an active role in the control and/or learning of unilateral hand movements. We approached this question by applying double-blind bihemispheric transcranial direct current stimulation (tDCS) over both contralateral and ipsilateral motor cortex in a between-group design during 4 d of unimanual explicit sequence training in human participants. Independently of whether the anode was placed over contralateral or ipsilateral motor cortex, bihemispheric stimulation yielded substantial performance gains relative to unihemispheric or sham stimulation. This performance advantage appeared to be supported by plastic changes in both hemispheres. First, we found that behavioral advantages generalized strongly to the untrained hand, suggesting that tDCS strengthened effector-independent representations. Second, functional imaging during speed-matched execution of trained sequences conducted 48 h after training revealed sustained, polarity-independent increases in activity in both motor cortices relative to the sham group. These results suggest a cooperative rather than competitive interaction of the two motor cortices during skill learning and suggest that bihemispheric brain stimulation during unimanual skill learning may be beneficial because it harnesses plasticity in the ipsilateral hemisphere.

**SIGNIFICANCE STATEMENT** Many neurorehabilitation approaches are based on the idea that is beneficial to boost excitability in the contralateral hemisphere while attenuating that of the ipsilateral cortex to reduce interhemispheric inhibition. We observed that bihemispheric transcranial direct current stimulation (tDCS) with the excitatory anode either over contralateral or ipsilateral motor cortex facilitated motor learning nearly twice as strongly as unihemispheric tDCS. These increases in motor learning were accompanied by increases in fMRI activation in both motor cortices that outlasted the stimulation period, as well as increased generalization to the untrained hand. Collectively, our findings suggest a cooperative rather than a competitive role of the hemispheres and imply that it is most beneficial to harness plasticity in both hemispheres in neurorehabilitation of motor deficits.

## Introduction

Even strictly unilateral motor behaviors such as moving one hand rely on interactions between the cerebral hemispheres. However, the nature of this interaction is incompletely understood (Perez and Cohen, 2009; Di Pino et al., 2014). One influential idea is the “interhemispheric competition” model, according to which the two hemispheres mutually suppress each other via inhibitory interconnections (Curtis, 1940; Ferbert et al., 1992; Daskalakis et al., 2002; Chen, 2004; Ni et al., 2009). This notion has shaped theories about how best to improve motor learning through brain stimulation. It has been suggested that motor learning may be facilitated, not only by exciting contralateral motor cortex, but also by inhibiting ipsilateral cortex, particularly in the context of stroke rehabilitation (Murase et al., 2004; Hummel and Cohen, 2006; Williams et al., 2010; Takeuchi et al., 2012). tDCS has been shown to increase motor-evoked potentials (MEPs) when the anode is placed above primary motor cortex (M1) (Nitsche and Paulus, 2000) and to decrease MEPs in the presence of a cathode (Nitsche et al., 2003). Consistent with the interhemispheric competition model, Vines et al. (2008) demonstrated that bihemispheric tDCS with the cathode located over ipsilateral M1 improves performance more than unihemispheric tDCS.

However, there are reasons to doubt that the bihemispheric advantage is due to the suppression of ipsilateral cortex. Although MEPs typically decrease under the cathode after unihemispheric stimulation (Nitsche et al., 2003), polarity-specific changes are reduced after bihemispheric stimulation (O'Shea et al., 2014). Therefore, increased neural plasticity subsequent to tDCS may not be closely linked to polarity-specific excitability changes measured with transcranial magnetic stimulation (TMS). Rather, plasticity increases may be attributable to the electrical current running transversely rather than radially through the cortical tissue (Rahman et al., 2013). If true, then tDCS effects should depend the spatial current distribution, not on current direction.

Under this assumption, bihemispheric tDCS may increase plasticity in both motor cortices independently of polarity. In addition, ipsilateral motor cortex may have an active role in the execution and learning of complex movements. Recent fMRI (Diedrichsen et al., 2013; Wiestler and Diedrichsen, 2013) and electrophysiological (Ganguly et al., 2009) studies have demonstrated that activity in M1, although often suppressed below baseline, contains information about ongoing ipsilateral movements. This activity could contribute to movement control either through directly descending ipsilateral projections or by shaping activation patterns on the contralateral side (Verstynen et al., 2005). According to this “interhemispheric cooperation” model, bihemispheric tDCS is more effective than unihemispheric stimulation, not because it silences ipsilateral cortex, but because it increases ipsilateral plasticity.

**Figure 1.**
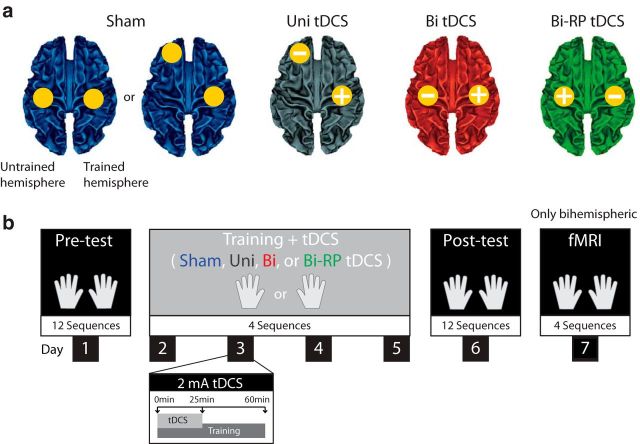
Experimental design. ***a***, tDCS montages: sham stimulation (blue); unihemispheric (“Uni”) tDCS (black); bihemispheric (“Bi”) tDCS (red); and RP bihemispheric (“Bi-RP”) tDCS (green). The location of the anode is indicated by a “+” and the location of the cathode by a “−”. Here, the electrode placement for the left-hand-trained group is shown (for which the right hemisphere was the “trained hemisphere”). For right-hand-trained participants, the electrode location was reversed for all stimulation groups. For purposes of double blinding, 2/3 of the sham group had a bihemispheric and 1/3 had an unihemispheric montage. ***b***, Procedure. During the pretest, participants performed 12 5-digit sequences with either hand. Subsequently, participants were assigned to 1 or 4 tDCS groups and trained for 4 d with either the left or the right hand, resulting in 8 different groups (for details, see Table 1). During the posttest, participants were tested again (as in the pretest) without tDCS. Finally, all participants with a bihemispheric montage (but not with unihemispheric montage) underwent fMRI scanning on day 7.

To adjudicate between these explanations, we tested the effects of reversing the polarity of bihemispheric stimulation. We trained 64 participants in a double-blind study using sham, unihemispheric, conventional bihemispheric, or reversed-polarity (RP) bihemispheric tDCS (Fig. 1*a*). Participants performed an unimanual sequence task with either the left or right hand over 4 d (Fig. 1*b*). After training and a behavioral posttest without tDCS, participants underwent fMRI to elucidate the neural changes induced by stimulation.

The interhemispheric competition model (assuming polarity-specific tDCS effects; see Table 1 for other assumptions) predicts that RP bihemispheric tDCS should impair performance compared with the sham group. Not only should contralateral cathodal stimulation suppress the motor areas involved in learning, but ipsilateral anodal stimulation should further increase interhemispheric inhibition. fMRI should reveal opposite changes in the hemisphere that received anodal and cathodal stimulation.

**Table 1.** Experimental prediction of hemispheric competition and cooperation models assuming either polarity specific or polarity unspecific effects

	Hemispheric competition model	Hemispheric cooperation model
Polarity-specific: anodal increases, cathodal decreases plasticity	Bihemispheric < unihemispheric < sham < bihemispheric-RP	Unihemispheric < bihemispheric ≤ bihemispheric-RP ≤ sham
Polarity-unspecific: both anodal and cathodal increase plasticity	Unihemispheric < bihemispheric = bihemispheric-RP < sham	Bihemispheric = bihemispheric-RP < unihemispheric < sham

Conditions are listed in order of predicted behavioral performance (< means lower movement time, i.e. better performance). Predictions for the hemispheric competition model assuming polarity-specific effects on plasticity and for the hemispheric cooperation model assuming polarity-unspecific effects are presented in the Introduction. Under the hemispheric competition model with polarity-unspecific tDCS effects, stimulating contralateral M1 only would be expected to be more effective than stimulating both (because both M1s are competing). However, both bihemispheric montages should still be more effective than sham as contralateral M1 is being stimulated. Under the hemispheric cooperation model with polarity-specific tDCS effects, unihemispheric tDCS is expected to have the greatest facilitatory effect because contralateral M1 is being stimulated and ipsilateral M1 is unaffected. The exact prediction for the remaining three conditions depends on the relative importance for each hemisphere in developing the skill.

In contrast, the interhemispheric cooperation model (assuming polarity-unspecific tDCS effects) predicts that plasticity is induced regardless of current direction and that the behavioral advantage arises from plasticity in both hemispheres. Therefore, RP tDCS should be as effective as conventional bihemispheric tDCS and both should be more effective than the unihemispheric montage. Given the bilateral nature of the predicted plastic changes, the tDCS-related advantages should generalize to the untrained hand. Finally, bihemispheric tDCS should lead to activity changes in both hemispheres in a polarity-unspecific manner.

## Materials and Methods

### 

#### 

##### Participants.

Sixty-four healthy, right-handed subjects (54.69% females; average age 22.84 ± 0.56 years) participated in this study. Participants completed the Edinburgh Handedness Inventory (Oldfield, 1971), as well as a survey of medical history, musical and computer gaming experience, and previous exposure to brain stimulation. Exclusion criteria for participation were as detailed previously (Waters-Metenier et al., 2014). Participants gave written informed consent in accordance with the Declaration of Helsinki and received financial remuneration with the possibility of additional bonuses for completing the study and performance improvements. The protocol was approved by the University College London Research Ethics Committee.

##### Sequence task.

The experiment required the fast production of different unimanual five-digit sequences. The sequences were performed on a custom-built, MRI-compatible, piano-like isometric force keyboard. Forces exerted on each key were measured every 5 ms via transducers (dynamic range of 0–25 N, FSG-15N1A; Honeywell).

The sequence task required participants to press each digit in a predefined order. A computer screen showed each sequence as a specific ordering of the numbers 1–5 where 1 referred to the left-most and 5 to the right-most key and the sequence was executed from left to right. In other words, sequences were cued in an extrinsic, spatial reference frame. Based on pilot experimentation (Wiestler and Diedrichsen, 2013), we selected 12 sequences of matched difficulty, excluding sequences that contained a run of more than three adjacent digits. From these 12 sequences, each participant was assigned a set of four sequences that would be trained (Wiestler et al., 2014). The possible sets for training were as follows: (1) 41352, 25314, 15423, and 51243; (2) 45132, 21534, 31425, and 35241; and (3) 52134, 14532, 23541, and 43125.

A small (0.53 cm × 0.53 cm) green box flanking the presented sequence was displayed on the left and right to cue the respective hand that should execute the sequence. A red box appeared on the other side indicating that the hand should remain at rest on the keyboard. The instructional stimulus remained on the screen for 2.7 s, after which 5 white asterisks were presented as a “go” signal. Each instructed sequence was executed either four times in a row (in the pretest and posttest) or three times (during training and fMRI acquisition) and each execution was individually triggered with a “go” signal. There was a 500 ms interval between consecutive sequence executions. Below, we define a single “trial” as the set of three or four consecutive executions of the same sequence.

The objective of the sequence task was to perform the five digit presses as quickly as possible with minimal errors. For a digit press to be registered, the active digit had to exceed exerting a force of 2.5 N, whereas all other digits had to generate forces of <2.2 N. After each digit press, the corresponding asterisk changed color to provide feedback about whether the individual press was as follows: “correct” (green), “incorrect” (red), or “too hard” (yellow; that is, greater than the upper limit set for the task, 8.9 N). Execution time (ET) was measured as the duration between onset of the first press and release of the last press and error rate was defined as the percentage of sequences that contained one or more incorrect digit presses. Throughout behavioral training, a constant error rate was encouraged by instructing participants to speed up if the error rate was lower than 20% and to slow down if it was higher.

After each sequence execution, participants saw a brief feedback (0.8 s): 1 green asterisk (1 point) indicated that all 5 presses of the sequence were executed correctly; 3 green asterisks (3 points) meant that the sequence was executed correctly and with ≥20% faster ET than the average of the previous run; 1 blue asterisk specified that the sequence was performed 20% slower than the average of the previous run (0 points); and 1 red asterisk signified that 1 or more errors were made (−1 point). Participants received a financial bonus according to their point score.

##### Experimental design.

All participants completed 4 study phases (Fig. 1): (1) a pretest (day 1) in which baseline performance for 12 5-digit sequences was evaluated for both hands; (2) a 4 d training phase (days 2–5) during which participants repeatedly practiced the same 4 sequences with either the left or right hand for approximately 1 h (which was coupled with 25 min of tDCS from the onset of training); (3) a posttest (day 6), which was conducted in the same manner as the pretest; and (4) an MRI session (day 7).

The pretest started with a short practice run with 4 trials or 16 executions (4 executions of 2 easy sequences per hand) to familiarize participants with the task. During the pretest, participants performed all 12 sequences (4 to-be-trained and 8 untrained) with both the left and right hands. Each hand was required to perform 2 trials per sequence with 4 executions per trial (i.e., 8 total executions). The pretest consisted of eight runs with 24 trials per hand, which resulted in a total of 96 executions per hand. Within the first four runs, the order of sequences and hands was permuted randomly and the order was reversed in the second half to counterbalance possible learning effects.

We assigned subjects pseudorandomly to one of four stimulation groups (sham, unihemispheric, bihemispheric, or RP bihemispheric tDCS groups) and to training cohort (left or right hand training) such that group differences in pretest performance were minimized (Waters-Metenier et al., 2014). ANOVA across tDCS groups revealed no difference in baseline task ET (*F*_(3,60)_ = 0.158, *p* = 0.923; see Table 2 for individual group means) and there was also no significant pairwise difference between any two stimulation groups (all *t* < 1.10, *p* = 0.273).

**Table 2.** Participant demographic and psychological variables

Group	Sham	Unihemispheric tDCS	Bihemispheric tDCS	Bihemispheric-RP tDCS	Over all 4 groups	
*N* (LH-trained; RH-trained)	21 (11; 10)	15 (8; 7)	14 (7; 7)	14 (7; 7)	Χ_(1)_^2^	*p*
					
Sex (Female: Male)	13: 8	7: 8	6: 8	9: 5	0.66	0.42

Detectability of tDCS status						
tDCS status: (Yes: No)	15: 6	11: 4	10: 4	11: 3	15:6	32:11
					Χ_(1)_^2^	*p*
					
					0.06	0.80

tDCS perception averaged across all training days					*F*_(3,60)_	*p*
Discomfort (0:10)	2.32 ± 0.46	2.72 ± 0.39	3.30 ± 0.54	2.88 ± 0.37	0.846	0.474
Perceived intensity (0:10)	2.81 ± 0.40	3.28 ± 0.44	2.84 ± 0.62	3.91 ± 0.41	1.164	0.331
Distraction due to tDCS (0:10)	1.07 ± 0.31	1.80 ± 0.38	1.63 ± 0.43	1.57 ± 0.39	0.825	0.485

Demographics					*F*_(3,60)_	*p*
Age	22.19 ± 0.95	24.00 ± 1.59	22.93 ± 0.99	22.43 ± 0.82	0.518	0.671
Handedness (Edinburgh)	88.09 ± 1.87	84.33 ± 4.57	77.5 ± 4.15	82.14 ± 3.95	1.661	0.185
Previous motor training (h)	640 ± 293	872 ± 396	447 ± 246	486 ± 306	0.336	0.800

Baseline motor performance					*F*_(3,60)_	*p*
Baseline ET_A_	2.36 ± 0.065	2.33 ± 0.15	2.45 ± 0.10	2.32 ± 0.069	0.159	0.924

Psychological measures					*F*_(3,60)_	*p*
Averaged across all days						
Sleep hours	7.29 ± 0.21	6.94 ± 0.22	7.49 ± 0.19	7.49 ± 0.21	1.393	0.254
Sleep quality (0:10)	7.28 ± 0.26	6.97 ± 0.19	7.89 ± 0.24	7.27 ± 0.26	2.155	0.102
Alertness (0:10)	6.77 ± 0.35	6.62 ± 0.51	7.21 ± 0.55	7.12 ± 0.24	0.413	0.744
Attention (0:10)	7.65 ± 0.28	7.56 ± 0.34	8.18 ± 0.32	7.04 ± 0.52	1.490	0.226
Task difficulty (0:10)	4.56 ± 0.45	5.43 ± 0.42	4.71 ± 0.37	5.16 ± 0.30	0.993	0.403
Averaged across final training day and posttest						
Semantic recall (% correct)	73.21 ± 4.59	88.33 ± 3.33	96.43 ± 1.57	93.75 ± 2.54	7.362	0.0003

To evaluate group difference differences in sex and detectability of tDCS status, all three tDCS groups were compared individually against sham using a χ^2^ goodness-of-fit test. For all other parameters, mixed-effects ANOVA was calculated with the between factor “tDCS.” All variables were averaged across left and right hand training cohorts.

At the beginning of each session, participants provided information about sleep quality, alertness, attention, and task difficulty using visual analog scales ranging from 0 (lowest) to 10 (highest). There were no significant differences between stimulation groups in terms of these parameters (Table 2).

To ensure that both participants and experimenter were blind to tDCS assignment, the randomization was performed by an investigator (J.D.) who was not involved in data collection. The experimenter (S.W.) only knew the hand training cohort and the electrode arrangement, not whether the participant received real or sham stimulation, which was determined by a randomized code entered into the tDCS machine at the beginning of each session. Accordingly, 66.7% of the sham participants (14/21) had bihemispheric electrode arrangement and 33.3% had unihemispheric electrode arrangement. No significant behavioral differences were found between these two subsets of the sham group on posttest performance (*F*_(1,16)_ < 1.454, *p* > 0.245; ANCOVA using pretest as covariate) for trained/untrained hand and trained/untrained sequence ET.

During the 4 training days, participants practiced 4 of the 12 sequences with either their left or right hand. A session consisted of 16 runs with 2 trials per sequence each. Therefore, participants performed 128 trials (384 sequence executions) per day. On the day after the final session of tDCS-coupled training, a posttest that had exactly the same structure as the pretest was administered.

##### tDCS.

tDCS was administered via a bihemispheric or unihemispheric montage. In the unihemispheric montage, we placed the anode over contralateral M1 and cathode over ipsilateral supraorbital ridge. For the bihemispheric montage, we positioned the anode over contralateral and the cathode above ipsilateral M1, with the RP montage involving the opposite polarity. The hand area of M1 was localized as the position where single-pulse suprathreshold TMS evoked a visible twitch in the contralateral first dorsal interosseus muscle (Boroojerdi et al., 1999). This was implemented using a Magstim BiStim2 with a 5 cm figure-of-eight coil positioned tangentially to the medial-sagittal plane skull at a 45° angle and with the handle pointing posteriorly and a monophasic pulse was delivered. This angle was chosen because it elicits the strongest the strongest perpendicular fields (Janssen et al., 2015), which is optimal for stimulating corticospinal neurons transsynaptically via horizontal corticocortical connections (Di Lazzaro et al., 2008; Delvendahl et al., 2014). tDCS was administered over 4 consecutive training days for the first 25 min of an ∼60 min session of sequence training. A current of 2 mA was delivered using a neuroConn DC-stimulator PLUS (http://www.neurocaregroup.com/dc_stimulator_plus.html) through saline-soaked 35 cm^2^ electrodes.

##### Behavioral analysis.

There was no significant difference between the tDCS groups in terms of error rate during the pretest (*F*_(3,60)_ = 0.252, *p* = 0.860; averaged across training group and hand) or training (*F*_(3,60)_ = 1.082, *p* = 0.364; averaged across day). We only found a significant effect of tDCS on error rate in the posttest, with tDCS recipients tending to be more accurate than sham (*F*_(3,60)_ = 3.086, *p* = 0.0339; averaged across training group and hand).

To adjust ET for the different error rates, we calculated the median ET for each run, sequence, and hand over all (correct and incorrect) trials. For this calculation, ET for the incorrect trial was replaced with the maximum ET of that group of trials, thereby penalizing inaccurate performance.

We conducted preplanned statistical comparisons between each stimulation group and sham, between bihemisperic and unihemispheric groups, and between the two bihemispheric groups for the last training day and the posttest. For these comparisons between pairs of groups, we used an ANCOVA in which the error-adjusted ET at pretest was used as a covariate to account for prior interindividual differences in performance. This procedure effectively subtracts from each training/posttest measurement the best prediction based on the pretest measurement. Compared with simply subtracting the pretest measurement, this method is less susceptible to noise induced by the larger variability in the pretest measurements. The ANCOVA included “tDCS group” and “hand training cohort” as between-subject factors. The threshold for all statistical comparisons was *p* < 0.05. All data presented in the text and figures are represented as mean ± SEM.

##### fMRI data acquisition.

All 42 bihemispheric tDCS recipients (sham and real) underwent fMRI scanning 1 d after the posttest. Due to resource limitations, we were not able to also scan the unihemispheric groups. Although this group would have supplied additional information, it was not critical to test our main hypothesis of polarity-independent changes in both hemispheres after bihemispheric stimulation. At this point, at least 48 h had elapsed since the final tDCS application. Therefore, any group differences should be due to long-term neuroplastic changes induced by tDCS because the immediate online effects of tDCS (Vernieri et al., 2010; Mielke et al., 2013; Antal et al., 2014) should have been washed out.

The fMRI session consisted of 8 runs comprised of 24 randomly ordered trials: 3 per trial type (4 sequences × 2 hands) with 3 sequence executions per trial, yielding 72 total executions per run. Each trial consisted of a cueing phase (2.7 s), followed by 3 executions of the cued sequence triggered 3.6 s apart. Therefore, a trial lasted 13.5 s. Each sequence execution had to be completed within 2.8 s to allow for a 0.8 s feedback phase.

To match behavioral performance during scanning, participants were instructed to produce the sequence at a fixed speed of 1.3 s and as accurately as possible. This ET was the fastest that subjects across all groups could achieve with both trained and untrained hands with high accuracy (∼90% correct). In addition, throughout training, force levels of the sequences were kept similar by imposing a maximal force threshold. No force level feedback was provided during fMRI scanning.

Baseline BOLD activation was measured during 8 randomly interspersed rest phases of 13.5 s. To monitor for mirror activity on the nonmoving hand, participants were required to keep all 10 digits on the keyboard and to generate a small baseline force of ∼0.5 N at all times.

Functional images were acquired using a 3 T Siemens Trio MRI machine with a 32-channel head coil. A 2D echoplanar sequence with a time of repetition (TR) of 2.72 s was used to acquire the functional volumes (8 runs, 159 volumes per run, 32 interleaved slices with 2.7 mm thickness) in an interleaved manner (3 mm gap and 2.3 × 2.3 mm^2^ in-plane resolution). The volumes were acquired in an oblique orientation with a 45° tilt angle from the AC–PC line; this slice prescription provided coverage of motor regions on the dorsal surface of the cortex, as well as the superior cerebellum and basal ganglia, but excluded inferior prefrontal and inferior and anterior temporal lobes. For full details of the fMRI acquisition, see Wiestler et al. (2014).

##### fMRI analysis.

The analysis of the fMRI data is described in detail in Wiestler et al. (2014), which reports the results from the sham group. After standard preprocessing (correction for slice acquisition, motion realignment, and coregistration to the individual anatomical image), we used a first-level linear model implemented in SPM8 (Friston et al., 2007) to estimate the activation for each of the four sequences for each hand. The design matrix consisted of a regressor for each hand and sequence type and an intercept for each run. The regressor was modeled as a boxcar function of 10.8 s beginning with the first go cue of the trial, which was then convolved with a standard hemodynamic response function. From the estimated regression weights, we computed the percentage signal change compared with rest for each hand averaged across the four sequences.

For the group analysis, we reconstructed the cortical surface of each individual participant using Freesurfer (Dale et al., 1999), which permits the extraction of the white–gray matter surface and pial surface from anatomical images. The functional data were then projected onto each individual surface by averaging for each surface node the voxels that lay between the white–gray matter and pial surfaces.

The individual surfaces were then aligned to a shared spherical template for left and right hemispheres. This allowed us to flip the results for the right-hand-trained cohort such that data from the trained hemisphere, the hemisphere contralateral to the trained hand, was displayed on the right group hemisphere and the data from the untrained hemisphere on the left. We then conducted *t* tests between sham and tDCS groups for each surface vertex using an uncorrected height threshold of *T*_(1,39)_ = 2.71, *p* = 0.005. Familywise error was controlled by calculating the critical size of the largest suprathreshold cluster that would be expected by chance using Gaussian field theory as implemented in the fmristat package (Worsley et al., 1996). Results were displayed using the 3D visualization software Caret (Van Essen et al., 2001).

For the profile plots shown in Figure 3, *a* and *b*, we defined a line running from the posterior parietal cortex through the hand area of M1 to the anterior tip of Brodmann's area 6 (BA6; premotor cortex). We then averaged activity over the area 1.5 cm above and below that line (purple area in Fig. 3*d*).

We also conducted a region of interest (ROI) analysis for the cortical motor regions. They included the hand region of primary motor cortex (M1, BA4); primary somatosensory cortex (S1, BA1–3); dorsal premotor cortex; supplementary and presupplementary motor areas; and superior parietal lobe, divided into an anterior (intraparietal sulcus) and posterior (occipitoparietal junction) aspect. We defined all ROIs on the symmetric group template and subsequently projected this into individual data space via the respective individual surface.

The average activity in these ROIs was submitted to a ANOVA to calculate differences between tDCS groups (factor “tDCS”) and interactions with training hand (factor “hand”) and hemisphere (factor “hemisphere”). Because there were no significant functional differences between left and right hand training cohorts (*F*_(1,36)_ < 2.21, *p* > 0.15, for all 6 ROIs, even without correction) or interactions with any other factor, we analyzed the functional data averaged over left and right hand training cohorts.

## Results

### Bihemispheric tDCS increases motor learning more than unihemispheric tDCS

**Figure 2.**
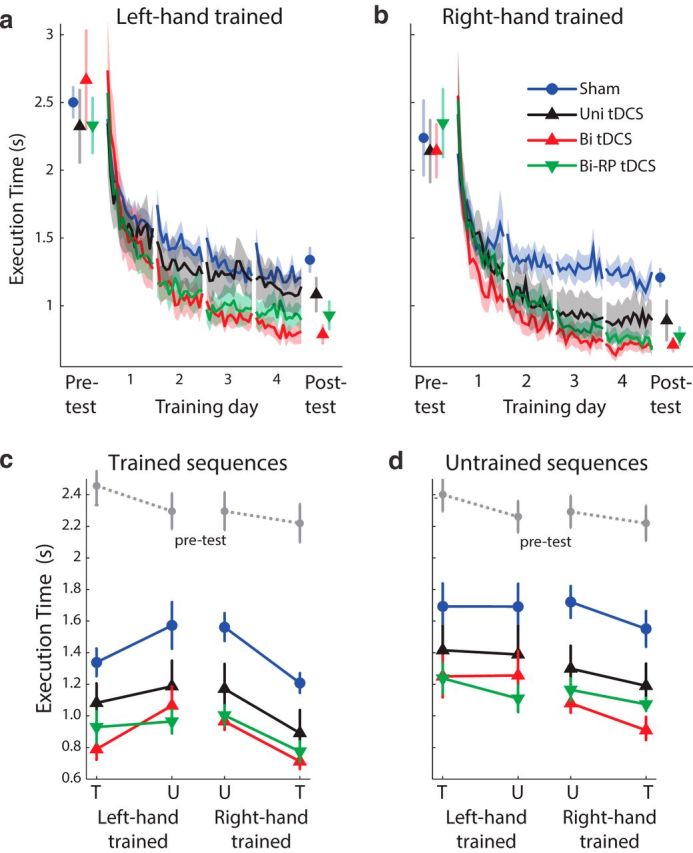
Bihemispheric tDCS accelerates learning and generalization in a polarity-independent manner. ***a***, ***b***, Average ET in the pretest, training, and posttest for sham (blue), unihemispheric (black), bihemispheric (red), and RP bihemispheric (green) tDCS groups. Subjects trained with either the left hand (***a***) or right hand (***b***). ***c***, ***d***, Pretest and posttest data for the trained (***c***) and untrained (***d***) sequences. Results are shown for the trained (T) and untrained (U) hands separated by group. The pretest results (gray dashed line) are averaged across all four groups. Error bars and shaded region indicate between-subject SEM.

We first determined whether conventional bihemispheric tDCS is more effective in promoting learning than unihemispheric stimulation. We used the error-adjusted ET (see Materials and Methods) as an overall performance measure. By the end of 4 d of sequence training, unihemispheric tDCS recipients executed sequences 0.203 ± 0.101 s (16.6%) faster than sham (*F*_(1,31)_ = 4.43, *p* = 0.043; Fig. 2*a*,*b*, black vs blue lines). As in previous reports (Reis and Fritsch, 2011), this advantage persisted during the posttest, which was conducted without tDCS 1 d after the final training session (*F*_(1,31)_ = 8.13, *p* = 0.008). There was no significant difference between the left- and right-hand-trained groups in the size of this effect (*F*_(1,31)_ = 2.03, *p* = 0.164).

Participants who received conventional bihemispheric tDCS were an additional 0.260 ± 0.114 s faster than those who received unihemispheric stimulation. The difference between bihemispheric and unihemispheric groups was reliable (*F*_(1,24)_ = 12.44, *p* = 0.002), as was the difference between bihemispheric and sham groups (*F*_(1,30)_ = 64.02, *p* = 6.24^−09^). These differences were maintained during the posttest (respectively, *F*_(1,24)_ = 10.98, *p* = 0.003 and *F*_(1,30)_ = 55.302, *p* = 2.75e^−08^). Therefore, moving the cathode from the supraorbital ridge (unihemispheric tDCS) to ipsilateral M1 (bihemispheric tDCS) yielded nearly twice the performance gain (37.8% relative to sham). Therefore, we replicate here higher effectiveness for bihemispheric tDCS relative to unihemispheric stimulation (Vines et al., 2008; Mahmoudi et al., 2011; Karok and Witney, 2013; Lindenberg et al., 2013; Sehm et al., 2013; Naros et al., 2016) in the context of a multiple-day learning study.

### RP bihemispheric tDCS increases motor learning

The interhemispheric competition model supposes that the advantage of bihemispheric relative to unihemispheric tDCS arises due to cathodal suppression of ipsilateral cortex. This idea predicts that the RP montage should attenuate motor learning relative to sham because it decreases contralateral M1 excitability both directly through cathodal and indirectly through ipsilateral anodal stimulation.

Our data, however, showed the converse: RP bihemispheric tDCS led to significantly faster ETs than sham stimulation, both during the final training day (*F*_(1,31)_ = 27.55, *p* = 1.154e^−05^) and during the posttest (*F*_(1,30)_ = 25.0, *p* = 2.32e^−05^). Statistically, the performance of the RP group was indistinguishable from conventional bihemispheric tDCS (last training day: *F*_(1,23)_ = 1.81, *p* = 0.191, posttest: *F*_(1,23)_ = 1.93, *p* = 0.178). The ETs for the conventional and RP bihemispheric groups were also symmetric across left- and right-hand-trained groups: the tDCS group × hand cohort interaction was not significant (*F*_(1,23)_ = 0.11, *p* = 0.7459). Even though RP bihemispheric tDCS led numerically to better outcomes than unihemispheric tDCS, this effect did not reach statistical significance (last training day: *F*_(1,24)_ = 2.97, *p* = 0.097, posttest: *F*_(1,24)_ = 2.02, *p* = 0.168). However, the two bihemispheric groups combined were significantly faster than the unihemispheric group (last training day: *F*_(1,38)_ = 8.61, *p* = 0.006, posttest: *F*_(1,24)_ = 6.66, *p* = 0.014).

Collectively, these results indicate that bihemispheric stimulation was more effective than unihemispheric stimulation. This additional benefit, however, was not conferred by ipsilateral suppression because we did not find a polarity-specific effect for bihemispheric tDCS. Instead, any stimulation of the ipsilateral motor areas accelerated motor learning.

These results, therefore, favor the bihemispheric cooperation model by which the additional learning advantage of bihemispheric relative to unihemispheric tDCS arises from plastic changes in both hemispheres that would promote performance for both hands. This idea makes two testable predictions. First, a considerable part of the behavioral tDCS advantage should generalize to the untrained hand. Second, neural changes should occur in both hemispheres in a polarity-unspecific manner. Below, we test these predictions.

### Behavioral tDCS effects generalize to the untrained hand

In the pretest and posttest, participants performed trained sequences with their untrained hand, allowing us to assess intermanual generalization. Consistent with previous results (Waters-Metenier et al., 2014; Wiestler et al., 2014), even the sham group showed considerable performance improvements on the untrained hand (Fig. 2*c*,*d*). This generalization was even more pronounced in the tDCS groups: relative to sham, unihemispheric (*F*_(1,31)_ = 12.43, *p* = 0.001); bihemispheric (*F*_(1,24)_ = 26.99, *p* = 1.34e^−05^) and RP bihemispheric groups (*F*_(1,24)_ = 25.98, *p* = 1.78e^−05^) all performed significantly better on the untrained hand. In addition, performance for the untrained hand was better in bihemispheric relative to unihemispheric tDCS recipients (*F*_(1,31)_ = 4.26, *p* = 0.047) and there was no difference between the bihemispheric tDCS groups (*F*_(1,23)_ = 0.05, *p* = 0.822).

Importantly, the enhancement of untrained hand performance was even larger than what would have been expected if generalization were simply proportional to the improvements on the trained hand: when expressing pretest/posttest difference for the untrained hand relative the learning gains for the trained hand, we found that tDCS increased the proportion of generalization. For sham recipients, the untrained hand gained 58.5% of the improvement of the trained hand, whereas, for both bihemispheric tDCS groups, the percentage of intermanual generalization was greater (86–89.2%; *t*_(1,33)_ > 2.662, *p* < 0.012). These results suggest that bihemispheric tDCS influenced mainly effector-independent representations in both hemispheres.

### Behavioral tDCS effects generalize to untrained sequences

All participants also improved on the untrained sequences from pretest to posttest (Fig. 2*d*). This effect was promoted by tDCS such that, for untrained sequences, unihemispheric tDCS recipients were 0.310 ± 0.105 s (19.4%) faster than sham and bihemispheric recipients (conventional and RP groups combined) were 0.231 ± 0.137 s (17.6%) faster than unihemispheric anodal tDCS recipients (and both of these differences were significant; respectively *F*_(1,31)_ = 6.73, *p* = 0.014 and *F*_(2,36)_ = 8.13, *p* = 0.008). In addition, when we quantified the proportion of transfer of speed advantages to untrained sequences for the trained hand, we found that, across all groups, tDCS increased the proportion of transfer relative to sham (*F*_(3,60)_ = 4.90, *p* = 0.0041). Therefore, the effect of tDCS in this study is exerted at a largely effector- and sequence-unspecific fashion, as we have reported previously (Waters-Metenier et al., 2014).

### Bihemispheric tDCS causes activation increases in both hemispheres

**Figure 3.**
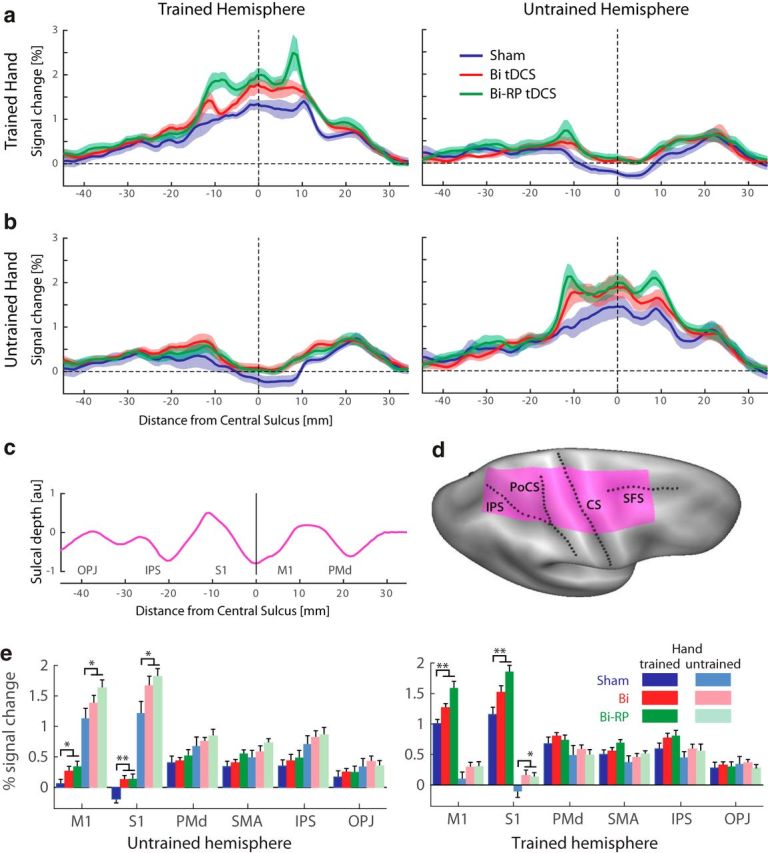
Bihemispheric tDCS recipients show greater average activation than sham in bilateral sensorimotor areas. ***a***, Profile plot of percentage signal change relative to rest in the trained and untrained hemispheres for trained hand executions. Results are shown for the sham, bihemispheric (Bi tDCS), and RP tDCS groups (Bi-RP tDCS). The *x*-coordinate indicates the distance from the fundus of the central sulcus along the cortical surface in millimeters running from occipital-parietal junction (posterior) to the rostral tip of premotor cortex (anterior). ***b***, Profile plot for the untrained hand. ***c***, Average sulcal depth along the profile. ***d***, Location of areas averaged in the profile plots on an inflated brain surface (purple area) ***e***, Average percentage signal change in six anatomically defined ROIs (Wiestler and Diedrichsen, 2013; Wiestler et al., 2014). Brackets indicate that the two bihemispheric groups were significantly different from sham. **p < 0.0083, statistical threshold for multiple comparisons, *p < 0.05. CS, Central sulcus; IPS, intraparietal sulcus; PoSC, postcentral sulcus; SFS, superior frontal sulcus.

**Figure 4.**
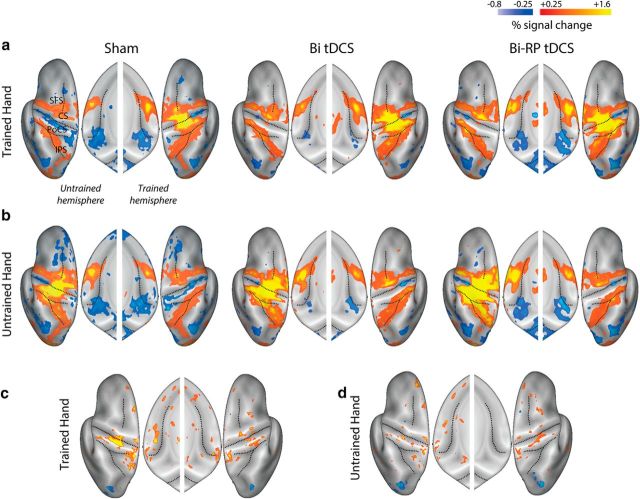
Bihemispheric tDCS recipients exhibit more activity in bilateral sensorimotor areas relative to sham. ***a***, Activation maps for the trained and untrained hemispheres for the sham, bihemispheric (Bi tDCS), and RP (Bi-RP tDCS) groups averaged across hand training cohort. Although the sham group exhibited ipsilateral deactivation (typically found in fMRI studies of unilateral movements), this pattern was not found for either bihemispheric tDCS group. In addition, the tDCS groups exhibited higher contralateral activation. ***b***, Corresponding maps for sequences executed with the untrained hand. ***c***, ***d***, Difference T-maps for the activation in the tDCS groups relative to sham for the trained (***c***) and untrained (***d***) hands. CS, Central sulcus; IPS, intraparietal sulcus; PoSC, postcentral sulcus; SFS, superior frontal sulcus.

To elucidate the neural consequences of tDCS stimulation, we measured fMRI BOLD activation while participants executed the four sequences with either the trained or untrained hand. Participants were scanned 2 d after their final tDCS-coupled training session such that our measure would reflect learning-related plasticity rather than immediate effects or aftereffects of tDCS on neural excitability or hemodynamics, which might not be directly related to sequence learning (Vernieri et al., 2010; Mielke et al., 2013; Antal et al., 2014). To prevent activity differences attributable to simple behavioral differences, we closely matched the performance in the scanner in terms of movement speed and force across both groups and hands. The hemispheric cooperation model predicts that similar neural changes should occur in both hemispheres independently of polarity. As expected, for trained hand executions, the sham group exhibited contralateral activation and ipsilateral deactivation in M1 and S1 (Figs. 3*a*, 4), as often observed during simple unimanual hand movements such as those that we studied here (Diedrichsen et al., 2013; Wiestler and Diedrichsen, 2013). In contrast, both bihemispheric tDCS groups, independently of polarity, showed greater contralateral activation and no ipsilateral deactivation.

For statistical comparison, we combined the two bihemispheric stimulation groups into a single tDCS group because we did not find any significant clusters of differential activation between conventional and RP tDCS groups. A surface-based group analysis (see Materials and Methods) showed that both contralateral and ipsilateral S1 and M1 cortex had greater activation in tDCS recipients (Table 3) for movements of the trained hand. Functional differences for the untrained hand were visually similar, but statistically less pronounced (Figs. 3*b*, 4*b*, Table 3).

**Table 3.** Effect of tDCS on average activation using surface-based analysis as tDCS > sham (% signal activation > rest)

Region (Brodmann's area)	Area (mm^2^)	Peak value *T*_(1,39)_	*p*_(clust)_	MNI coordinates		
				*x*	*y*	*z*
Trained hand						
Contralateral (trained) hemisphere						
Postcentral (S1; BA3)	145.33	3.64	0.007	49.89	−22.22	51.78
Postcental (S1; BA3)	116.21	3.81	0.030	18.26	−39.58	65.61
Precentral (M1; BA4)	114.01	3.78	0.033	37.77	−20.16	57.22
Ipsilateral (untrained) hemisphere						
Precentral (M1; BA4)	574.01	5.11	<0.001	−37.41	−26.36	55.81
Postcentral (S1; BA3)	229.65	4.45	<0.001	−17.03	−39.31	69.61
Orbital area/pars triangularis (BA47)	182.63	5.49	0.002	−42.46	29.98	−0.78
Untrained hand						
Contralateral (untrained) hemisphere						
Orbital area/pars triangularis (BA47)	116.31	4.39	0.031	−41.68	30.01	−0.44
Ipsilateral (trained) hemisphere = all nonsignificant						

Between-subject analysis with uncorrected height threshold of *T*_(1,39)_ = 2.71, *p* = 0.005. Area indicates the size of the suprathreshold cluster, *T*_(1,39)_ the maximal *t*-value, *p*_(clust)_ is the corrected probability of observing a cluster of this size or bigger over the whole cortical surface by random chance (Worsley et al., 1996). Coordinates reflect the location of the cluster peak in MNI space.

Analysis using anatomically predefined ROIs (Wiestler and Diedrichsen, 2013) confirmed these results (Fig. 3*e*). Across both hemispheres and hands, we observed activation increases in M1 (*F*_(1,40)_ = 9.34, *p* = 0.004) and S1 (*F*_(1,40)_ = 13.55, *p* = 0.0007). There was some evidence for an interaction of tDCS × hemisphere × hand in S1 (*F*_(1,40)_ = 4.16, *p* = 0.048), although this did not quite reach significance in M1: *F*_(1,40)_ = 3.72, *p* = 0.061. This reflects that bihemispheric tDCS especially increased activation associated with the trained hand and ipsilateral (untrained) hemisphere.

We also observed the expected hemispheric asymmetries with more ipsilateral activity in the left hemisphere during left hand movements compared with activity in the right hemisphere during right hand movements (Verstynen et al., 2005). This hemispheric asymmetry was significant for M1 (*F*_(1,39)_ = 31.137, *p* = 1.983e^−6^) and S1 (*F*_(1,39)_ = 21.73, *p* = 3.621e^−5^), but did not interact significantly with the tDCS effect.

Despite our best efforts to match behavioral performance during fMRI, there were slight, but significant differences between the groups (Table 4). However, the differences in ET were very small (∼40–60 ms) and the differences in force relative to sham were not consistent across the two bihemispheric tDCS groups (force was slightly lower than sham in the conventional bihemispheric tDCS group and slightly higher than sham in the RP tDCS group). Nevertheless, to ensure that the observed increases in activation could not be attributable to these small behavioral differences, we included execution time, error rate, and force as covariates in ANCOVA analyses and found that, for all comparisons, the effect of tDCS remained significant for both M1 (respectively, *F*_(1,39)_ = 7.26, *p* = 0.01; *F*_(1,39)_ = 14.03, *p* = 0.001; *F*_(1,39)_ = 8.29, *p* = 0.006) and S1 (*F*_(1,39)_ = 8.02, *p* = 0.007; *F*_(1,39)_ = 10.99, *p* = 0.002; *F*_(1,39)_ = 12.78, *p* = 0.001).

**Table 4.** Execution time, error rate, and force during fMRI

	Sham		Bihemispheric tDCS		RP-bihemispheric tDCS		ANOVA across groups	
	Mean	SE	Mean	SE	Mean	SE	*F*_(2,39)_	*p*
Trained hand								
Execution time (s)	1.36	(0.03)	1.30	(0.02)	1.32	(0.02)	2.602	0.0869
Error rate (%)	13.76	(2.36)	5.08	(1.18)	5.61	(1.70)	7.206	0.0022
Force (N)	5.99	(0.34)	5.65	(0.29)	6.85	(0.22)	4.627	0.0157
Untrained hand								
Execution time (s)	1.40	(0.04)	1.32	(0.02)	1.31	(0.01)	4.506	0.0174
Error rate (%)	15.43	(2.03)	6.50	(1.23)	5.75	(1.30)	11.864	0.0001
Force (N)	5.69	(0.26)	5.50	(0.27)	6.71	(0.29)	5.718	0.0066

Table shows mean (±SE) of behavioral parameters for all three tDCS groups during fMRI scanning (averaged across hand training cohort). The last column indicates an *F*-test comparing the three groups.

Another potential confound that could lead to increased ipsilateral activation is mirroring. Mirroring refers to the phenomenon whereby muscles of the nonmoving hand are activated simultaneously with those of the moving hand (Beaulé et al., 2012). Such movements are typically visible in pathological states (e.g., stroke), but are also present and measurable in healthy populations. During fMRI, participants were required to rest the passive hand on the keyboard while the active hand was executing sequences. Mirroring was parameterized using the range of forces on the passive hand across the time course of a trial, the associated SD, and the correlation between the force traces for matching digits of the passive and active hand. Even though the significant positive correlations indicated that we could successfully detect the very subtle mirroring in our healthy control participants, there were no significant difference between tDCS groups for any of these measures (Table 5).

**Table 5.** Mirroring of digit forces during fMRI

	Sham		Bihemispheric tDCS		RP-bihemispheric tDCS		ANOVA across groups	
	Mean	SE	Mean	SE	Mean	SE	*F*_(2,39)_	*p*
Trained hand								
Force range	0.13	(0.01)	0.14	(0.01)	0.15	(0.03)	0.356	0.703
SD	0.03	(0.00)	0.03	(0.00)	0.04	(0.01)	0.497	0.612
Correlation of force traces	0.07	(0.002)	0.07	(0.05)	0.03	(0.03)	0.387	0.682
Untrained hand								
Force range	0.12	(0.01)	0.12	(0.01)	0.13	(0.02)	0.293	0.745
SD	0.03	(0.00)	0.03	(0.00)	0.03	(0.01)	0.260	0.773
Correlation of force traces	0.09	(0.02)	0.05	(0.03)	0.02	(0.03)	1.981	0.154

Table shows mean (±SE) of the average force range in Newtons (maximum − minimum) and SD of the passive (nonmoving) hand. Results were split depending on whether the trained hand or the untrained hand was the passive hand. The correlation between the forces was calculated between the force time series of the matching digits of the active and passive hands. The last column indicates the *F* test comparing the three groups.

Together, these data demonstrate that bihemispheric tDCS, regardless of the polarity, was associated with increases in average activation in both ipsilateral and contralateral hemispheres. This difference could not be explained by behavioral differences between the groups during the scan.

### Quantification of subject blindedness and perceptual side effects of tDCS

**Figure 5.**
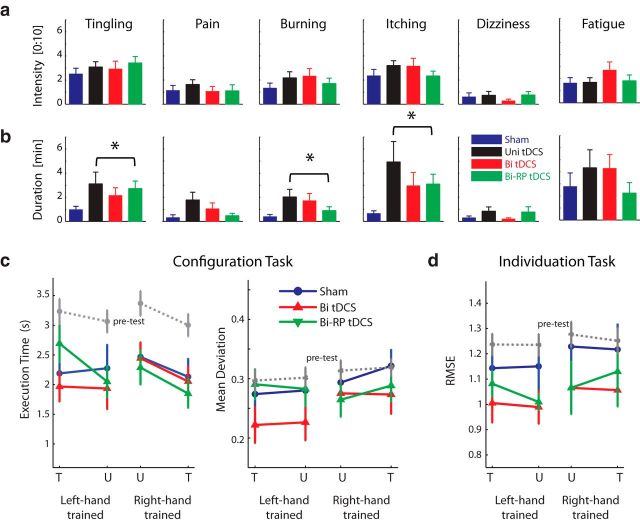
Online perceptual side effects of tDCS. We monitored the potential online side effects of tDCS to evaluate perceptual differences between tDCS groups and sham. ***a***, For the intensity level of all six effect types, there were no significant differences across tDCS groups. ***b***, However, the differences between tDCS and sham tended to be greater for the duration of these side effects and significant differences were observed for duration of burning and itching (indicated by asterisks). ***c***, Posttest performance of the configuration task speed (left) and mean deviation (right) for the sham (blue), bihemispheric (red), and RP bihemispheric (green) groups. ***d***, Posttest performance of individuation root mean square error. tDCS recipients experienced no adverse behavioral effects as a result of tDCS-coupled sequence training.

Subsequent to each session of tDCS administration, participants underwent a previously designed battery to characterize tDCS effects (Waters-Metenier et al., 2014) with the addition of questions about perceptual side effects. Specifically, participants were asked to rate the experience of tingling, pain, burning, itching, dizziness, and mental fatigue (1:10) and then to describe (in minutes) the duration of the effect. As can be seen in Figure 5*a*, tDCS recipients tended to exhibit slightly higher intensities of perceptual properties of tDCS, especially in terms of tingling, burning and itching; however, none of these variables showed a significant between-group difference (all *t*_(62)_ < 1.446, *p* > 0.153) or exceeded level 4 (of a maximal rating of 10) for any of the 64 subjects.

However, the groups tended to differ on how long they experienced these side effects (Fig. 5*b*). Averaged across the three tDCS groups (unihemispheric, bihemispheric, and RP bihemispheric), subjects reported significantly longer durations of tingling (*t*_(62)_ = 2.553, *p* = 0.013), burning (*t*_(62)_ = 2.492, *p* = 0.015), and itching (*t*_(62)_ = 2.833, *p* = 0.006) and a tendency for longer duration of pain (*t*_(62)_ = 1.806, *p* = 0.076). There were no differences in the duration of dizziness (*t*_(62)_ = 1.051, *p* = 0.298) or mental fatigue (*t*_(62)_ = 0.671, *p* = 0.505) between sham and tDCS groups. As observed previously (Waters-Metenier et al., 2014), participants experienced no significant differences in overall discomfort, perceived tDCS intensity, or distraction due to tDCS (Table 2). Moreover, χ^2^ goodness-of-fit tests showed that there were no differences in the detectability of tDCS assignment between tDCS groups and sham (Table 2). These findings collectively suggest that, despite slight perceptual differences, tDCS blinding methods were sufficient. This is congruent with other recent evidence (Kessler et al., 2012), including a study that investigated over twice as many subjects at 2 mA (Russo et al., 2013).

### Assessment of behavioral side effects of tDCS

In addition to the main training task (the sequence task described above), we tested all bihemispheric tDCS and sham recipients on two additional tasks of manual skill during the pretest and posttest: individuation (the ability to move the digits separately) and configuration execution (the skill of pressing certain digits at the same time while keeping coactivation of unintended digits minimal; for full details of these tasks, see Waters-Metenier et al., 2014). Neither bihemispheric tDCS group exhibited any side effects from tDCS-coupled sequence training on either of these skills (Fig. 5*c*,*d*) and there were no significant differences between the two bihemispheric groups and sham (all *F*_(2,35)_<2.092, *p* > 0.139; using ANCOVA correction with pretest performance). Therefore, as in our previous work, we did not find any adverse trade-offs between using tDCS to facilitate manual motor skill learning and performance on untrained tasks (Waters-Metenier et al., 2014).

## Discussion

Our study provides evidence for an active role of ipsilateral motor regions in unimanual motor skill learning. We replicated the classic observation that bihemispheric tDCS is more effective than stimulating only one hemisphere (Vines et al., 2008). Critically, bihemispheric tDCS with the anode over ipsilateral motor cortex led to similar learning advantages as with the anode over contralateral motor cortex. Finally, both montages led to long-lasting increases of functional activity in bilateral sensorimotor areas and the tDCS-induced learning advantage generalized to the untrained hand.

Our results clearly argue against the interhemispheric competition model as an explanation for the advantage of bihemispheric over unihemispheric tDCS. According to this idea, cathodal stimulation suppresses the ipsilateral hemisphere, subsequently releasing the contralateral hemisphere from interhemispheric suppression. Our results for the RP bihemispheric group are at odds with this explanation because excitatory stimulation of the ipsilateral cortex should have led to attenuated, rather than accelerated, motor learning relative to unihemispheric (or even sham) stimulation.

Instead, the full pattern of our results can be explained under the assumption that tDCS increased plasticity in both hemispheres independently of polarity and that the two motor cortices cooperate in producing high levels of skill (see Table 1). One limitation of our study is that we did not measure changes in bilateral MEPs directly before and after tDCS. Therefore, we cannot make strong inference about whether the cathodal stimulation in the bihemispheric montage increased or decreased short-term excitability in M1. For example, there is some evidence that cathodal unihemispheric tDCS stimulation at 2 mA increases, rather than decreases, MEPs (Mordillo-Mateos et al., 2012; Batsikadze et al., 2013; but see Cengiz et al., 2013). Importantly, previous studies of the effect of bihemispheric tDCS on MEPs have not exhibited consistent excitability changes using a bihemispheric montage: in contrast to unihemispheric tDCS, bihemispheric stimulation has been found to either produce no significant changes in MEPs (O'Shea et al., 2014) or changes that were statistically less robust (Mordillo-Mateos et al., 2012). These results raise the possibility that the changes in motor plasticity shown behaviorally in this and previous studies (Vines et al., 2008) rely on different mechanisms than those reflected in the polarity-specific changes in MEPs. A parsimonious explanation for our results is that the behavioral tDCS effects are related to the spatial distribution of electrical currents rather than to the current direction. Biophysical current modeling of tDCS (Truong et al., 2014; Naros et al., 2016) demonstrates that the unihemispheric montage primarily sends current through contralateral premotor and ipsilateral prefrontal regions, whereas bihemispheric stimulation targets motor and premotor regions bilaterally. Importantly, the weak radial currents that give rise to the polarity-specific effects on MEPs switch directions on opposite sides of the gyrus (Rahman et al., 2013). Therefore, premotor areas in both hemispheres could experience either suppression or excitation depending on their folding geometry. The effects of tDCS on neuroplasticity, therefore, could be mediated by the considerably stronger tangential currents, which, in contrast to the radial currents, do not have polarity-specific effects (Rahman et al., 2013). Under this assumption, bihemispheric tDCS (independently of polarity) would have increased neural plasticity in both hemispheres in a manner unrelated to the changes measureable with MEPs.

Even under the assumption of polarity-unspecific tDCS effects on plasticity, our results remain incompatible with the hemispheric competition model: if the cathode promoted plasticity in ipsilateral M1 during training, then the interhemispheric competition model would have predicted a disadvantage of both bihemispheric montages relative to the unihemispheric montage because bihemispheric stimulation would increase the putatively harmful ipsilateral activation (see Table 1).

Instead of probing excitability of the primary motor cortex after tDCS stimulation using TMS, we evaluated task-related activity using fMRI after transient tDCS effects had been washed out. An advantage of this approach is that we avoided possible interference with the process of memory consolidation through the necessary TMS stimulation to M1 when measuring MEPs (Muellbacher et al., 2002). We observed that the average activity during trained hand movements was larger in bihemispheric tDCS groups in both contralateral and ipsilateral sensorimotor regions. The results for the untrained hand were similar, albeit less robust. Previous work has demonstrated bilateral activity increases in M1 during the application of bihemispheric tDCS (Lindenberg et al., 2013). Similar online (Kwon and Jang, 2011; Stagg et al., 2012) or short-lasting (Baudewig et al., 2001; Kwon et al., 2008; Jang et al., 2009; Stagg et al., 2009; Kim et al., 2012) changes underneath the anode have also been reported for unihemispheric tDCS. Importantly, we measured functional activity ∼48 h after the end of the final stimulation. Therefore, the activity increases reported here reflect longer-lasting changes caused by neural plasticity rather than any immediate effects of tDCS on the hemodynamic response.

The fact that the activation changes in our study were restricted to M1 and S1 should not necessarily be taken as evidence that the relevant neuroplastic changes only occurred here. Rather, it is equally possible that tDCS led to increased plasticity bilaterally in premotor or supplementary motor cortex and that the increased activity in M1 and S1 reflects the increased modulatory input from these areas.

Increased plasticity in motor and premotor areas of both hemispheres would also explain our observation that the behavioral advantages due to tDCS generalized to the untrained hand. In a previous study, we used multivoxel pattern analysis to identify commonalities in the neural encoding of specific motor sequences for the left and right hands (Wiestler et al., 2014)^.^ We found widespread, shared representations in both premotor and, surprisingly, primary motor areas of both hemispheres. Given that tDCS increased the amount of intermanual generalization, it appears likely that effector-independent sequence representations were particularly facilitated.

To summarize, we demonstrate that conventional and RP bihemispheric tDCS similarly increase motor learning and BOLD activation in both anode- and cathode-modulated hemispheres. Therefore, our study provides evidence for an active role of the ipsilateral cortex in unimanual motor control in young, healthy individuals, consistent with previous reports in older adults or victims of stroke (Johansen-Berg et al., 2002; Zimerman et al., 2014). tDCS effects on plasticity are still often construed in terms of excitation/inhibition of neural tissue under the electrodes and the interhemispheric competition model is commonly used to explain the superiority of bihemispheric tDCS. The idea of promoting motor learning by reducing ipsilateral excitability is particularly pertinent to stroke neurorehabilitation, where it has led to the supposition that suppressing activity in the healthy hemisphere to release inhibition of lesioned cortex may facilitate recovery (Hummel and Cohen, 2006; Williams et al., 2010; Bolognini et al., 2011; Nitsche and Paulus, 2011; Takeuchi and Izumi, 2012; Zimerman et al., 2012; Krause et al., 2013; Lefebvre et al., 2014). Although the hemispheric cooperation model proposed here must be tested in elderly participants and individuals with stroke, our data suggest that a simple excitation/inhibition model may be too simplistic and should be abandoned in favor of a framework that acknowledges the broad effects of tDCS current and the roles that both hemispheres play in the encoding of information during motor learning.
